# Improving the ostrich genome assembly using optical mapping data

**DOI:** 10.1186/s13742-015-0062-9

**Published:** 2015-05-12

**Authors:** Jilin Zhang, Cai Li, Qi Zhou, Guojie Zhang

**Affiliations:** 1 China National GeneBank, BGI-Shenzhen, Shenzhen,, 518083 China; 2 Centre for GeoGenetics, Natural History Museum of Denmark, University of Copenhagen, Øster Voldgade 5-7, 1350 Copenhagen, Denmark; 3 Department of Integrative Biology, University of California, Berkeley, USA; 4 Department of Biology, Centre for Social Evolution, University of Copenhagen, Universitetsparken 15, 2100 Copenhagen, DK Denmark

**Keywords:** Ostrich, Optical mapping, Genome assembly

## Abstract

**Background:**

The ostrich (*Struthio camelus*) is the tallest and heaviest living bird. Ostrich meat is considered a healthy red meat, with an annual worldwide production ranging from 12,000 to 15,000 tons. As part of the avian phylogenomics project, we sequenced the ostrich genome for phylogenetic and comparative genomics analyses. The initial Illumina-based assembly of this genome had a scaffold N50 of 3.59 Mb and a total size of 1.23 Gb. Since longer scaffolds are critical for many genomic analyses, particularly for chromosome-level comparative analysis, we generated optical mapping (OM) data to obtain an improved assembly. The OM technique is a non-PCR-based method to generate genome-wide restriction enzyme maps, which improves the quality of *de novo* genome assembly.

**Findings:**

In order to generate OM data, we digested the ostrich genome with *Kpn*I, which yielded 1.99 million DNA molecules (>250 kb) and covered the genome at least 500×. The pattern of molecules was subsequently assembled to align with the Illumina-based assembly to achieve sequence extension. This resulted in an OM assembly with a scaffold N50 of 17.71 Mb, which is 5 times as large as that of the initial assembly. The number of scaffolds covering 90% of the genome was reduced from 414 to 75, which means an average of ~3 super-scaffolds for each chromosome. Upon integrating the OM data with previously published FISH (fluorescence *in situ* hybridization) markers, we recovered the full PAR (pseudoatosomal region) on the ostrich Z chromosome with 4 super-scaffolds, as well as most of the degenerated regions.

**Conclusions:**

The OM data significantly improved the assembled scaffolds of the ostrich genome and facilitated chromosome evolution studies in birds. Similar strategies can be applied to other genome sequencing projects to obtain better assemblies.

## Data description

The advent of the next-generation sequencing (NGS) technology (e.g. Illumina HiSeq, SOLID, 454 FLX) has facilitated the new genome sequencing projects. However, the short reads produced by NGS limits the *de novo* assembly process to overcome the repeat-rich or highly heterozygous regions to obtain long scaffolds. Without long scaffolds, it is difficult or impossible to conduct some downstream analyses, such as chromosomal rearrangement analysis. One good method used to elongate the scaffolds is optical mapping (OM) [1], which estimates the gap length between scaffolds and merges them into much longer sequences without introducing new bases.

The flightless ostrich (*Struthio camelus*) is the tallest and heaviest living bird. It is the only member in the family Struthionidae, which is the basal extant member of Palaeognathae. Ostrich meat is considered healthy due to its high polyunsaturated fatty acid content, low saturated fatty acid content, and low cholesterol level. The worldwide production of ostrich meat is around 12,000 to 15,000 tons per year [2]. Due to this bird’s biological and agricultural importance, the avian phylogenomics project sequenced the ostrich genome for phylogenetic [3] and comparative genomics analyses [4]. Because ostrich is an important species for avian chromosome evolution analysis [5,6], we generated OM data to help improve the assembly.

To increase scaffold lengths with OM technology, the input genome assembly must meet certain requirements as follows: (1) the minimum scaffold N90 should be ≥200 kb and (2) N% in the genome should be <5%. Our Illumina-based assembly fully met these requirements. Before generating OM data, a series of restriction enzymes was evaluated based on the average DNA fragment size produced. This enabled us to check their compatibility with and coverage in the ostrich genome (Table 1). To determine the best enzyme, numerous criteria were applied to define their feasibility, including the percentage of usable DNA fragments within a certain size range, maximum fragment size, number of fragments generated, *etc*. (Table 1). After evaluation, we chose *Kpn*I as the most efficient enzyme for the ostrich genome for use in subsequent experiments.

**Table 1:** **Restriction enzymes evaluated for compatibility with the Ostrich genome**

Enzyme	Usable % 5-20 kb	Usable % 6-12 kb	Usable % 6-15 kb	#Frags >100 kb	Avg. frag. size (kb)	Max. frag. size (kb)
***Afl*** **II**	11.46	4.78	4.60	1	3.90	67.77
***BamH*** **I**	95.20	86.52	77.07	6	8.00	127.15
***Kpn*** **I**	96.77	87.02	62.77	14	10.39	148.23
***Nco*** **I**	63.32	34.18	33.67	0	4.81	74.89
***Nhe*** **I**	88.66	63.85	63.26	0	6.19	84.12
***Bgl*** **II**	0.99	0.36	0.36	0	3.08	36.92
***Spe*** **I**	69.60	33.80	32.55	1	5.66	105.87
***Xba*** **I**	12.20	4.63	4.46	0	3.95	59.07

All work done in this project followed the guidelines and protocols for research on animals and had the necessary permits and authorization. High molecular weight genomic DNA was extracted from a blood sample collected from a male ostrich in the Kunming Zoo of China. The DNA was then transferred to OpGen, Inc. for collection of single molecule restriction maps (SMRMs) on the Argus® Whole Genome Mapping System. The average size of the digested molecules was ~282 kb, which was determined to be sufficient. To further confirm the enzyme compatibility and performance, 3 MapCards were run to examine the average fragment size, the results of which were consistent with the expected outcome.

In total, 32 high-density MapCards were collected and ~136,000 molecules were marked for each card. Finally, about 1.99 million molecules (>250 kb) were analyzed using Genome-Builder (Table 2), OpGen’s analysis pipeline for restriction map comparison. Briefly, *in silico* restriction maps were first generated from the Illumina assembly based on the *Kpn*I recognition site. These maps were then used as seeds to find overlaps with the SMRMs obtained from the DNA molecules by map-to-map alignment in the Genome-Builder pipeline. Overlapped maps were then assembled with the *in silico* maps to produce elongated maps, where low coverage regions towards both ends were discarded to maintain the high confident extensions. In our study, we performed four iterations to ensure sufficient extensions. In each iteration, the extended scaffolds were used as the seeds for the next iteration. The extended scaffolds were then used to perform pairwise alignment. The resulting alignments that passed the empirical confidence threshold were considered candidates to connect scaffolds. The relative location and orientation of each of the pairs of the connected scaffolds were used to generate super-scaffolds. This elevated the assembly quality and achieved a scaffold N50 of 17.71 Mb, which is 5 times as large as the scaffold N50 of the initial assembly (Table 3).

**Table 2:** **Summary of SMRM data**

	All	Maps of >250 kb
**Total size**	1,126,357.03 Mb	732,483.56 Mb
**Number of molecules**	3,925,195	1,989,698
**Average molecule size**	286.96 kb	368.14 kb
**Minimum molecule size**	150.11 kb	250 kb
**Average fragment size**	16.854 kb	17.598 kb

**Table 3:** **Summary of assemblies**

	Scaffold N50	Scaffold N90	N%	Total size
**Initial assembly**	3.59 Mb	561Kb	3.30	1.26Gb
**OM assembly**	17.71 Mb	3.41 Mb	5.56	1.23Gb

**Figure 1:**
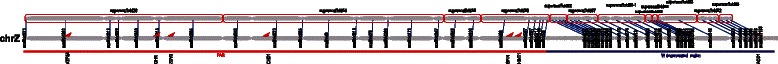
Relationships between OM super-scaffolds and the Illumina assembly scaffolds. The upper part of the figure shows the super-scaffolds generated by OM, and the lower shows the ordered Illumina scaffolds by aligning against the chicken Z chromosome. Because we made use of the FISH markers (red triangles) to resolve the artificial rearrangements introduced by alignment with the chicken genome, the scaffold order of the lower part was not the original order from the whole genome alignment. The red and blue underlines represent the PAR and W degenerated region, respectively.

To demonstrate that OM assembly can facilitate chromosome evolution research, we present an example of the Z chromosome. Together with previously published FISH (fluorescence *in situ* hybridization) markers [7], OM makes it possible to re-organize and anchor the scaffolds to the relevant position on the Z chromosome. We recovered the PAR (pseudoautosomal region) by jointing 4 super-scaffolds and their corresponding FISH markers (Figure 1). It is worth mentioning that upon OM integration with FISH markers, most of the sequences in the W degenerated region were properly placed (Figure 1). The longest super-scaffold anchored to the ostrich Z chromosome is 29.2 Mb. Considering the gap sequence introduced by OM could not elucidate more information on the whole Z chromosome, we ignored the gap size estimated from OM and filled in a constant gap of 600 Ns between scaffolds. This avoided introducing more uncertainty into the sequence and simplified the downstream analysis. The pseudo Z chromosome we constructed further extended our knowledge of evolutionary strata and their diversity in birds, making it possible to deduce the rearrangement events during different periods [8]. In addition, together with the multi-genome alignments, we further examined the force of Z chromosome evolution in birds [9].

In conclusion, the OM data generated in this study and presented here improved the ostrich assembly and facilitated a comparative analysis at the chromosome level. The improved assembly can be used for future genomic studies, especially those requiring long scaffolds. Furthermore, these data can be used for future development of OM software tools.

## Availability of supporting data

The data files presented in this Data Note are available in the *GigaScience* repository, GigaDB [10]. Raw sequencing data are also available from the SRA [SRP028745].

## Abbreviations

OM: Optical mapping
SMRM: Single molecule restriction map
FISH: Fluorescence *in situ* hybridization
PAR: Pseudoautosomal region
